# Biomarker Profiling for Pyridoxine Dependent Epilepsy in Dried Blood Spots by HILIC-ESI-MS

**DOI:** 10.1155/2018/2583215

**Published:** 2018-08-01

**Authors:** Elizabeth Mary Mathew, Sudheer Moorkoth, Leslie Lewis, Pragna Rao

**Affiliations:** ^1^Department of Pharmaceutical Quality Assurance, Manipal College of Pharmaceutical Sciences, Manipal Academy of Higher Education, Manipal 576104, India; ^2^Department of Paediatrics, Kasturba Medical College, Manipal Academy of Higher Education, Manipal 576104, India; ^3^Department of Biochemistry, Kasturba Medical College, Manipal Academy of Higher Education, Manipal 576104, India

## Abstract

Pyridoxine dependent epilepsy is a condition where the affected infant or child has prolonged seizures (status epilepticus), which are nonresponsive to anticonvulsant therapy but can be treated with pharmacological doses of pyridoxine. If identified earlier and treated prophylactically with pyridoxine, severe brain damage due to seizures can be prevented. Alpha-amino adipic semialdehyde (AASA), piperidine-6-carboxylic acid (P6C), and pipecolic acid (PA) are known biomarkers of pyridoxine dependent epilepsy. We report the development and validation of a hydrophilic interaction liquid chromatography (HILIC) hyphenated with mass spectroscopy for the quantification of the above analytes from dried blood spot samples. The samples were extracted using methanol and analysed on a iHILIC fusion plus column with formic acid buffer (pH 2.5): acetonitrile (20:80) at a flow rate of 0.5 mL/min within 3 minutes. The method demonstrated a LOD of 10 ng/mL, LOQ of 50 ng/mL, linearity of r^2^ ≥ 0.990, and recovery of 92-101.98% for all analytes. The intra- and interday precision CVs were < 8% and 6%, respectively. Extensive stability studies demonstrated that the analytes were stable in stock solution and in matrix when stored at -80°C. We performed method comparison studies of the developed method with the literature reported method using normal samples and matrix matched spiked samples at pathological concentrations to mimic clinical validity. The Bland-Altman analysis for comparison of the analytical suitability of the method for the biomarkers in healthy and spiked samples with the literature reported method revealed a bias which suggested that the method was comparable. The newly developed method involves no derivatisation and has a simple sample preparation and a low run time enabling it to be easily automated with a high sample throughput in a cost-effective manner.

## 1. Introduction

Pyridoxine dependent epilepsy (PDE) is an autosomal recessive metabolic encephalopathy that presents in affected newborns within the first month of life with myoclonic, tonic clonic seizures in the neonatal period and partial seizures in the early infantile period. These seizures are known to respond to pyridoxine and reoccur on pyridoxine withdrawal [1, 2]. The exact incidence of PDE is still unknown. Studies conducted in Netherlands, United Kingdom, and Ireland report the incidence as 1:100000 to 700000 individuals and more than 100 cases have been reported worldwide [3, 4]. This disorder arises due to the mutation in the antiquitin gene which codes for the alpha-amino adipic semialdehyde dehydrogenase enzyme that is responsible for the conversion of alpha-amino adipic semialdehyde to alpha-amino adipic acid. Defect in the enzyme leads to the accumulation of alpha-amino adipic semialdehyde (*α*-AASA) that exists in equilibrium with piperidine-6-carboxylic acid (P6C). The formed P6C undergoes Knoevenagel condensation with pyridoxal phosphate in the body leading to its deficiency. P6C is also known to be an intermediate in the pipecolic acid pathway and its accumulation leads to enzymatic block which in turn results in toxic levels of pipecolic acid [5].

Analytical platforms like LC-MS have been explored for the quantification of AASA, P6C, and PA in various body fluids [6–11]. These methods employ derivatisation using fluorenylmethyloxycarbonyl chloride or butanolic hydrogen chloride [12] in order to alter the retention on C18 columns. Recently the hyphenation of hydrophilic interaction liquid chromatography with mass spectrometry have demonstrated suitability in the quantification of pipecolic acid in plasma [11], but reports on the suitability of this chromatographic technique for AASA or P6C are unknown. At present the diagnosis of this disorder involves the quantification of these biomarkers from plasma, serum, and urine samples. A recent investigation has demonstrated the suitability of dried blood spots as a suitable matrix for these analytes especially for infants and newborn [9]. The objective of the current study was to develop and validate a novel derivatisation free and sensitive bioanalytical LC-MS method for simultaneous quantification of AASA/P6C and PA using HILIC-ESI-MS from dried blood spot samples.

## 2. Materials and Methods

### 2.1. Materials and Reagents

L-allysine ethylene acetal (AEA) (>98%), Amberlyst 15 hydrogen form, and pipecolic acid (PA) (99%) were purchased from Sigma-Aldrich (St. Louis, US). DL-pipecolic acid-d9 (internal standard) was purchased from Toronto Research Chemicals (Ontario, Canada). LC-MS grade acetonitrile was purchased from Biosolve Chimie SARL (Dieuze, France). Formic acid, 85% (AR grade), was purchased from Merck (Kenilworth, US). In-house Milli Q water (Siemens Ultra Clear) was used. iHILIC fusion (+) column (100 × 2.1 mm, 3.5 *μ*m) was purchased from HILLICON AB (Umea, Sweden).

### 2.2. Instrumentation

A Thermo Scientific (Massachusetts, US) LC-mass spectrometer with Dionex Ultimate 3000 liquid chromatograph interfaced with a linear ion trap analyser by an electron spray ionisation source was used. MS/MS and chromatographic method development was performed using LTQ XL (Massachusetts, US) and Chromeleon (Massachusetts, US) software, respectively. Batch analysis was done using the XCalibur software (Massachusetts, US) and quantification was done using LC Quan (Massachusetts, US).

### 2.3. Sample Collection

Ethical clearance (MUEC/010/2017 dated 08.05.2017) was obtained from the Ethics Committee of Manipal Academy of Higher Education, Manipal. Dried blood spot samples were collected from neonates in Kasturba Hospital, Manipal, Karnataka, India, for a period of six months from July 2017 by a certified nurse. The samples were dried at room temperature for three hours followed by which the samples were transferred to envelopes with desiccants. The envelopes were then transferred to zip lock plastic bags and were stored at -80°C until further analysis.

### 2.4. AASA and P6C Synthesis

Due to the lack of a true reference standard for AASA, we proceeded to the synthesis of AASA in the laboratory based on reported methods [8] with some modifications. 5 mg AEA and 15 mg Amberlyst beads were mixed in 1 mL of water for 10 min using a shaker at 1320 rpm. The resultant solution was filtered and the remaining Amberlyst beads were washed with 0.5 mL water and transferred to the same filter. The washing and filtering steps were repeated with 1 mL of water twice. Deblocking efficiency was checked by comparing the mass spectra obtained before and after reaction. The obtained solution was further diluted with 3.1 mL water. The MS intensities observed for AASA and P6C were 1:3. The reproducibility of the procedure was checked by multiple injections using different stock preparations. The resultant stock solution had a concentration of 1 mmol/L of AASA and 3 mmol/L of P6C. This solution was used for the preparation of calibrators and controls. From this stock solution, a working stock containing 0.3*μ*g/mL of AASA and 1 *μ*g/mL of P6C (henceforth referred to as equilibrium mixture in the manuscript) was prepared for optimizing MS parameters by direct infusion.

### 2.5. Calibrators and Quality Controls

The calibrators and quality controls samples were prepared in leftover blood from healthy controls after adjusting the haematocrit to 50% similar to that of a newborn. Stock solutions of PA, d9-PA (1000 *μ*g/mL), and AASA/P6C (321 *μ*g/mL) were prepared in water. Working stocks were prepared in 50:50%v/v acetonitrile: water and spiked into aliquots of haematocrit adjusted blood. 40*μ*l of the spiked blood was pipetted onto Whatman 903 filter paper to prepare calibrators and quality controls (LLQC, LQC, MQC, and HQC) as per Table 1.

### 2.6. LC-MS Method and Sample Analysis

**Figure 1:**
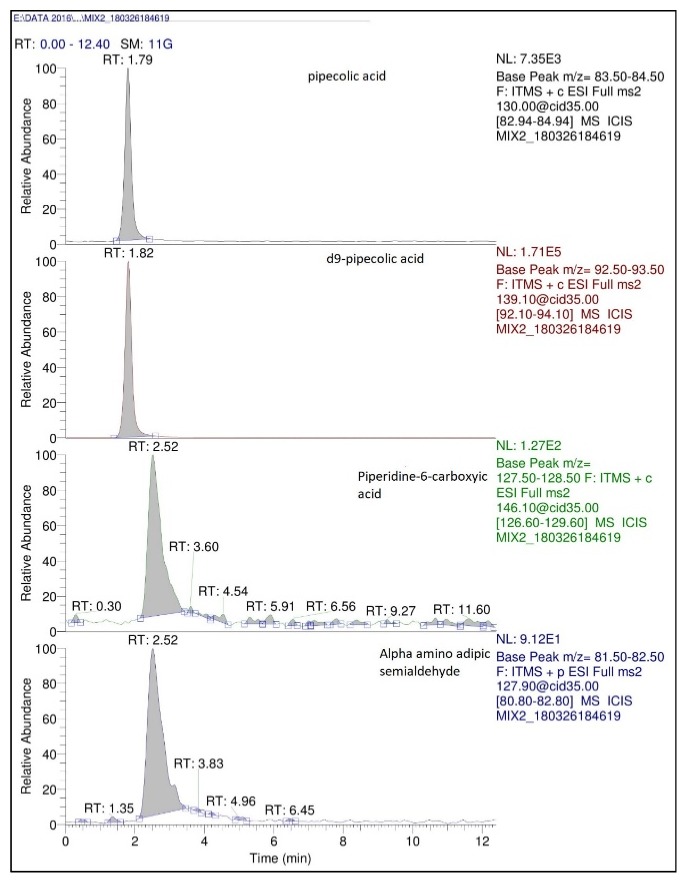
Optimized LC-MS chromatogram of *α*-amino adipic semialdehyde, piperidine-6-carboxylic acid, and pipecolic acid in SRM mode.

Optimization of MS conditions was performed by infusing solutions of PA (1 *μ*g/mL) and the equilibrium mixture (0.3 *μ*g/mL AASA and 1 *μ*g/mL P6C) independently at a rate of 10 *μ*L/min through the direct infusion pump in ESI (+) polarity mode. During optimization, the mass scan filters were set at a centre mass of m/z 128, 146, 130, and 139 for P6C, AASA, PA, and d9-pipecolic acid, respectively with a width of m/z 10. The iHILIC fusion (+) column facilitated the retention of PA and AASA/P6C without derivatisation. The mobile phase consisted of 80 volumes of acetonitrile and 20 volumes of formic acid buffer (pH 2.5) delivered isocratically at a flow rate of 0.5 mL/min. The sample injection volume was optimized at 10*μ*L. The retention times of PA, AASA/ P6C, and d9-PA were 1.79, 2.59, and 1.80 minutes, respectively. The total run time of the method was 3 minutes. The optimized mass spectrometer parameters were as follows: spray voltage: 5V, vaporizer temperature: 300°C, nitrogen sheath gas flow: 55 arbitrary units, auxiliary gas flow: 12 arbitrary units, sweep gas flow: 2 arbitrary units, ion transfer capillary temperature: 275°C with a voltage of 15.00 V and tube lens: 90 V, multipole 00 offset: -5.75 V, lens 0: -9.50V, multipole 0 offset: -9.75V, lens voltage: -15V, gate lens: -70,00V, multipole 1 offset: -15.50 V, multipole RF amplitude: 400 V, and front lent lens: -14.75 V. The optimized SRM transition for each analyte was as follows: AASA (*m/z* 146 → 128.13, CE: 35), P6C (*m/z* 127.9 →81.8, CE: 35), PA (*m/z* 130 → 83.94, CE: 40), and d9-PA (*m/z* 139 → 93.1 CE: 40) in SRM mode. The optimized chromatogram is presented in Figure 1.

### 2.7. Extraction Optimization from Dry Blood Spot (DBS)

3.2 mm DBS were made using standard leather punch and transferred to a 0.5 mL Eppendorf tube followed by the addition of 100*μ*L of methanol containing the internal standard. The tubes were vortex mixed for 30 min and the resultant solutions were evaporated to dryness at 30°C using a nitrogen evaporator for 5 min. The dried residue was reconstituted in the mobile phase and the subsequent solution was injected to LC-MS.

For the quantification of AASA/P6C and pipecolic acid in the dried blood spot, the method reported by Yuzyuk [12] was adopted. A ten-point calibration curve was constructed based on the peak area ratio of the analyte to internal standard (d9-pipecolic acid). Since AASA and P6C exist in equilibrium in the body, the calibration curve of AASA/P6C was constructed based on the sum of the area ratios of AASA and P6C on the Y axis and concentration on the X axis. Each batch consisted of blank (mobile phase), zero standard (with internal standard), a set of ten calibrators, and QC samples (bracketed and placed at regular intervals among the unknown samples).

### 2.8. Method Validation

Validation studies were performed as per Food and Drug Administration (FDA) guidelines [13]. A batch of six DBS samples from healthy neonates was processed with internal standard to assess the specificity of the method. To determine the LOD and LOQ we followed the procedure of Gachet et al. [14]. The LOD was determined using standard solutions serially diluted until a signal to noise ratio (S/N) of 3 was obtained. LLOQ was determined using six spiked DBS samples processed with the internal standard at which the S/N was 10 and showed positive values after blank subtraction. Linearity, accuracy, and recovery were determined by the blank subtraction technique [14, 15]. To perform linearity, endogenous levels of the analyte in the matrix were first determined by the standard addition method and this endogenous analyte to IS ratio was subtracted from the spiked DBS analyte to IS ratio. The subtracted peak area ratio was plotted versus analyte concentration and regression analysis was performed. Five replicates at each concentration for PA and AASA/P6C were performed for linearity. The accuracy of the method was determined by comparing the mean calculated responses of six replicate QC samples (after subtraction of endogenous level) to the nominal concentration values at each level. The absolute extraction recoveries of analytes were determined by comparing the responses (after subtraction of endogenous level) of six replicates at LQC, MQC, and HQC levels with neat standard solutions of same concentrations prepared in the mobile phase. The matrix effect was determined by comparing the response (after subtraction of endogenous level) obtained by the postextraction spike method at LQC and HQC levels (six replicates) with corresponding standard solutions at each QC level prepared in the mobile phase. The carry-over was measured using blank samples injected after analysis of the upper limit of quantification (ULOQ) samples with internal standard. Intraday and interday precision were performed in two batches by analysing six replicates at four QC levels on a single day and over three separate days, respectively.

### 2.9. Stability Studies

Extensive stability studies for the biomarkers at different conditions recommended by the guidelines were performed [13]. Stock solution stability was performed by comparing six replicates of a freshly prepared neat MQC with that of the stability samples at 2, 4, 6, and 8 h at room temperature. Stability of analytes in matrix was evaluated using six replicates of LQC and HQC at bench top (0, 0.5, 1, 1.5, 2, 4, and 7 h, room temperature) and freeze thaw (3 cycles, -80°C) and in autosampler (6, 12, 24, and 48 h at 4°C). Long-term stability on storage at -80°C was assessed on 7, 15, 30, 60, 90, 180, and 360 days using six replicates of LQC and HQC. On the day of the analysis samples were thawed unassisted at room temperature and compared with initial results at each QC level. The results obtained were computed for mean, standard deviation, and percent change. The results obtained were analysed by “repeated measures ANOVA” and “paired t-test”. A p value < 0.05 was considered statistically significant.

### 2.10. Method Comparison Studies

Method comparison study was performed to assess how the newly developed method correlates with the method reported in literature. 50 samples were coded, and a blind analysis was carried out on the developed HILIC method. These DBS samples were then subjected to analysis by the method reported by Yuzuk et al. [12]. The results were analysed by Bland-Altman analysis using SPSS 16.0 for method comparison.

Since there was no true clinical sample available to demonstrate the clinical validity of the assay, we followed the approach recommended by Taverniers et al. [16] and Jung et al. [9]. To mimic positive samples, we first determined the endogenous levels of the analytes in blood by the standard addition approach and eleven spiked DBS samples at pathological levels (matrix matched) were prepared by a separate analyst at levels of 1-6*μ*g/mL and 0.5-3 *μ*g/mL for AASA/P6C and pipecolic acid, respectively, based on reports of Sadilkova et al. [8]. All the samples were coded with numbers and a blind random analysis was conducted by a separate analyst on five different days on the developed HILIC method and by the method reported by Yuzuk et al. [12]. The result obtained was analysed by Bland-Altman analysis using SPSS 16.0.

## 3. Results and Discussion

PA, AASA, and P6C are intermediates in the catabolism of lysine. These analytes are polar in nature and their retention on reverse phase columns is challenging. We have explored the ability of hydrophilic interaction liquid chromatography and LC-MS for the determination of these analytes in dried blood spot. Three HIILIC columns, namely, Venusil amide (100 X 4.6 mm, 3*μ*), iHILIC fusion (100 X 2.1 mm, 3*μ*), and iHILIC fusion plus (100 X 2.1 mm, 3*μ*), were investigated for their suitability at pH 2.5 and 4.5 using 90 parts of acetonitrile and 10 parts of buffer. At pH 4.5 the iHILIC column and the iHILIC fusion plus column demonstrated a capacity factor greater than 15 for all the analytes. At pH 2.5 the iHILIC fusion plus demonstrated a capacity factor of less than 8. Hence the iHILIC fusion plus column at pH 2.5 was seen to be suitable for further experimental studies. The retention times of PA, AASA/P6C, and d9-PA were 1.79, 2.52, and 1.82 min, respectively (Figure 1). The ability of mass spectrometry for selective reaction monitoring (SRM) enabled us to achieve unique advantage in terms of specificity. The MS^2^ function in linear ion traps enabled different SRM transitions for different analytes (PA:* m/z* 130 → 83.94, CE: 40; d9-PA:* m/z* 139 → 93.1, CE: 40). These differences in SRM transitions enabled the quantification of the analytes even though there was coelution in chromatography.

The DBS extraction procedure was optimized for extraction solvent, rotation speed (rpm), and rotation time. 100% methanol was seen to produce maximum recovery. An increasing trend in recovery was observed with increasing the rotation time with no further increase after 30 min at 400 rpm. The method was seen to be cost-effective and demonstrated recoveries of 90.33-92.16 % and 80.22-85.12% for PA and AASA/P6C, respectively (Table 1).

The lower limit of detection and limit of quantification was 10 ng/mL (S/N of 3) and 50 ng/mL (S/N of 10), respectively for all the analytes. No significant endogenous interferences were observed at the retention time of the analytes and ISTD proving the method to be specific. The method demonstrated good linearity (0.05-10 *μ*g/mL) with regression coefficient (r^2^) value at or above 0.997. Our LC-MS method achieved good precision with a total CV of 3.12-7.8% (AASA/P6C) and 1.6- 5.0% (PA) at the various quality control levels. The accuracy of the method was evaluated through spike recovery and ranged from 92.06 to 101.98 % and 98.10 to 100.87 % for AASA/P6C and PA, respectively, at various quality control levels. The validation summary is provided in Table 1.

**Figure 2:**
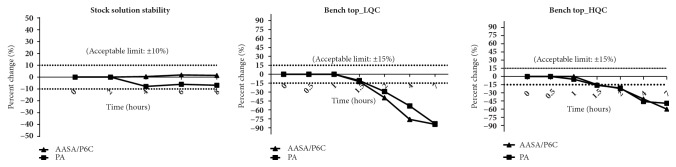
Stock solution stability studies and bench top stability studies of low quality controls (LQC) and high-quality controls (HQC) of *α*-amino adipic semialdehyde, piperidine-6-carboxylic acid, and pipecolic acid.

**Figure 3:**
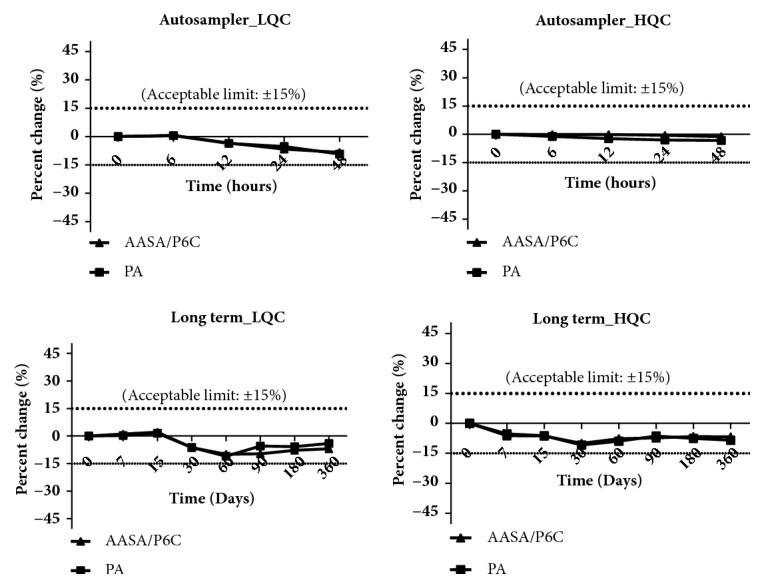
Autosampler and long-term stability studies of low quality controls (LQC) and high-quality controls (HQC) of *α*-amino adipic semialdehyde, piperidine-6-carboxylic acid, and pipecolic acid.

Results of the stability studies represented by mean back calculated concentrations and the respective % changes are as presented in Tables 2 and 3. PA and AASA/P6C were found to be stable in stock solution prepared in methanol with a percent change of 1.43% and -6.87 %, respectively, at eight hours from the baseline value (Table 2 and Figure 2). These levels are well within the recommended limit of ±10%. The bench top stability studies undertaken at LQC and HQC levels for each analyte to study the influence of laboratory conditions demonstrated a decreasing trend in the levels of the analytes with time. We observed a % change of -12.44% and 10.73% from initial value at 1.5 h for AASA-P6C and pipecolic acid, respectively, at LQC levels. Additionally, at HQC levels we observed a % change of -15% from baseline for AASA-P6C and -16.13% for pipecolic acid at 1.5 h. More than 80% change from baseline value was observed within 7 h. Our results align parallel to the earlier reports by other workers in urine and serum [6, 8, 9]. To study the influence of freeze thaw cycles on analyte integrity, we measured the basal levels of AASA/P6C and PA at LQC and HQC levels. The second measurement was made after subjecting the QC's to three freeze thaw cycles. AASA/P6C demonstrated stability for three cycles with a percent change of -6.3% and -2.45% at LQC and HQC levels, respectively. At LQC levels PA demonstrated a percent change of -7.15% and at HQC it was -0.53%. These observations are well within the acceptance limit of ±15% denoting stability. The stability study on the influence of resident time in the autosampler maintained at 4°C at LQC levels demonstrated a percent change of -8.35% and -9.23 % within 48 hours for AASA/P6C and PA, respectively. At HQC levels these changes were -1.23% and -3.3%. Long-term stability studies at -80°C for a period of 360 days demonstrated a change of only -6.96 % and -4.05 % at LQC levels and a change of -6.77 % and -8.43 % at HQC levels for AASA/P6C and PA, respectively. These results are within the recommended limit of ± 15, indicating long-term stability for these analytes similar to earlier reports in plasma [8]. Graphical representations of the stability data are presented in Figures 2 and 3.

**Figure 4:**
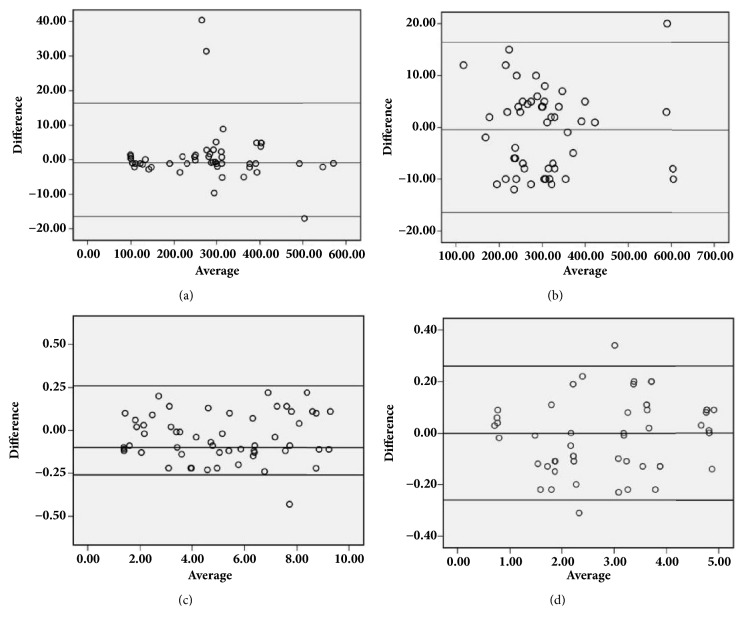
Bland-Altman analysis of the developed HILIC-MS method with literature reported LC-MS method for healthy and matrix matched spiked samples. (a) Healthy: *α*-amino adipic semialdehyde/piperidine-6-carboxylic acid. (b) Healthy: pipecolic acid. (c) Matrix matched spiked: *α*-amino adipic semialdehyde/piperidine-6-carboxylic acid. (d) Matrix matched spiked: pipecolic acid.

Results of the comparison study of the developed LC-MS method to the literature reported method showed that the mean ± SD for normal sample was 279.64 ±120.90 and 306.12 ±128.88 ng/mL by the developed HILIC-MS method compared to 280.92 ±121.77 and 307.85 ±128.22 ng/mL for AASA/P6C and PA, respectively, by the literature reported method. The Bland-Altman analysis (Figure 4) revealed a bias of -0.99 and -1.91 for AASA/P6C and PA, respectively, proving that the methods are comparable.

The study conducted to mimic the clinical validity using spiked positive control samples demonstrated a recovery of 82-106% and 92.8-119% for AASA/P6C and pipecolic acid, respectively. Additionally the Bland-Altman analysis of the results of % recovery using SPSS 16.0 showed a mean±SD of 3.40±1.62 and 1.94±0.84 *μ*g/mL by the developed HILIC-MS method compared to 3.44±1.62 and 1.95±0.81 *μ*g/mL for AASA/P6C and pipecolic acid, respectively, by the literature reported method with a bias of -0.04 and -0.10 for AASA-P6C and pipecolic acid, respectively (Figure 4).

## 4. Conclusions

A simple, rapid, and sensitive method for the simultaneous determination of AASA/P6C and pipecolic acid has been developed and validated on HILIC-ESI-MS/MS. The method has a low run time and lacks derivatisation. These advantages permit us to handle a large sample throughput in a cost-effective manner. Further work in this direction can be undertaken to confirm the clinical validity of the method with true clinical samples of PDE.

## Figures and Tables

**Table 1:** Method performance specifications for AASA/P6C and pipecolic acid.

**Analyte**	**Performance specifications**								
	**Calibration range (** ***μ*** **g/mL)Linearity**	**LOQ (** ***μ*** **g/mL)**	**LOD (** ***μ*** **g/mL)**	**QC Levels (** ***μ*** **g/mL)**	**Accuracy (**%**)**		**Precision (**%**)**		**Percent Recovery (**%**)**
					**Inter**	**Intra**	**Inter**	**Intra**	
AASA/P6C	0.05-10 (r^2^=0.999)	0.05	0.01	LLOQ (0.05)	92.06	99.71	3.43	3.12	-
				LQC (0.5)	101.98	100.15	5.16	5.31	80.22
				MQC (1)	100.78	96.78	3.60	4.16	80.88
				HQC (8)	101.32	94.12	7.87	5.12	85.12

Pipecolic acid	0.05-9 (r^2^=0.997)	0.05	0.01	LLOQ (0.05)	100.10	99.22	2.25	3.78	-
				LQC (0.5)	98.10	100.87	1.66	5.00	90.33
				MQC (1)	100.32	100.21	3.38	2.84	93.23
				HQC (8)	98.17	98.74	2.34	2.31	92.16

LOQ: limit of quantification, LOD: limit of detection, LLOQ: lower limit of quantification, LQC: low quality control, MQC: medium quality control, HQC: high quality control, AASA: alpha-amino adipic semialdehyde, and P6C: piperidine-6-carboxylic acid.

**Table 2:** Results of stock solution stability for alpha-amino adipic semialdehyde/piperidine-6-carboxylic acid and pipecolic acid.

Stock solution stability				
	AASA/P6C		PA	
Time points (hours)	Mean back calculated concentration (SD) (mcg/mL)^1^	% change	Mean back calculated concentration (SD) (mcg/mL)^1^	% change
0	0.89 (0.04)	0.00	0.85 (0.01)	0.00

2	0.89 (0.06)	0.05	0.85 (0.01)	0.00

4	0.90 (0.01)	0.45	0.78 (0.03)	-7.84

6	0.91 (0.1)	1.79	0.80 (0.03)	-6.10

8	0.91 (0.007)	1.43	0.79 (0.03)	-6.87

P value^2^	0.138		0.007	

^1^Values expressed as mean (SD); ^2^statistical treatment performed using repeated measures ANOVA. p value < 0.05 was considered significant. AASA: alpha-amino adipic semialdehyde, P6C: piperidine-6-carboxylic acid, and PC: pipecolic acid.

**Table 3:** Results of bench top, freeze thaw, autosampler, and long term stability for alpha-amino adipic semialdehyde/piperidine-6-carboxylic acid and pipecolic acid.

**Stability expressed as “mean back calculated concentration” (** ***μ*** **g/mL)** ^1^ **± SD,**				
**(**%** change)**				
**Bench top stability (room temperature, 7 hours)**				

Time point	LQC		HQC	
	AASA/P6C	Pipecolic acid	AASA/P6C	Pipecolic acid

0 hr	0.44±0.01 (0.00)	0.44±0.01 (0.00)	7.45±0.10 (0.00)	7.54±0.05 (0.00)

0.5 hr	0.44±0.01 (0.00)	0.43±0.00 (0.00)	7.21±0.31 (0.00)	7.49±0.14 (0.00)

1 hr	0.44±0.01 (0.00)	0.43±0.00 (0.00)	7.16±0.21 (0.00)	7.15±0.21 (-5.21)

1.5 hr	0.38±0.02 (-12.44)	0.38±0.01 (-10.73)	6.40±0.02 (-15.00)	6.39±0.21 (-16.13)

2 hr	0.26±0.02 (-39.40)	0.31±0.02 (-29.11)	5.80±0.20 (-22.20)	5.91±0.16 (-21.19)

4 hr	0.10±0.01 (-75.91)	020±0.02 (-53.33)	4.36±0.55 (-41.43)	4.04±1.29 (-46.17)

7 hr	0.06±0.01 (-84.20)	0.07±0.008 (-83.30)	2.91±0.94 (-60.00)	3.79±0.15 (-49.37)

p value^2^	<0.05	<0.05	<0.05	<0.05

**Freeze thaw stability (three freeze thaw cycles)**				

0 cycle	0.44±0.01 (0.00)	0.44±0.01 (0.00)	7.45±0.11 (0.00)	7.50±0.05 (0.00)

3 cycles	0.41±0.02 (-6.31)	0.41±0.02 (-7.15)	7.27±0.38 (-2.45)	7.46±0.06 (-0.53)

p value^3^	0.11	0.08	0.36	0.36

**Autosampler stability (4**°**C, 48 hours)**				

0 hr	0.45±0.01 (0.00)	0.44±0.01 (0.00)	7.75±0.10 (0.00)	7.50±0.05 (0.00)

6 hr	0.46±0.01 (0.59)	0.45±0.01 (0.52)	7.74±0.11 (-0.14)	7.41±0.14 (-1.15)

12 hr	0.44±0.01 (-3.39)	0.43±0.01 (-3.61)	7.74±0.14 (-0.16)	7.33±0.21 (-2.31)

24 hr	0.42±0.01 (-6.65)	0.42±0.01 (-5.24)	7.71±0.13 (-0.50)	7.28±0.17 (-3.01)

48 hr	0.42±0.03 (-8.35)	0.48±0.03 (-9.23)	7.65±0.14 (-1.23)	7.25±0.16 (-3.30)

p value^2^	0.05	0.04	0.68	0.06

**Long term stability (-80**°**C, 360 days)**				

0 day	0.45±0.01 (0.00)	0.44±0.01 (0.00)	8.47±0.20 (0.00)	7.18±0.23 (0.00)

7 days	0.46±0.02 (1.13)	0.44±0.02 (0.28)	7.95±0.49 (-6.16)	6.80±0.42 (-5.30)

15 days	0.46±0.03 (2.10)	0.45±0.02 (1.57)	7.94±0.10 (-6.22)	6.73±0.12 (-6.21)

30 days	0.43±0.02 (-6.24)	0.42±0.02 (-6.14)	7.63±0.06 (-9.96)	6.40±0.08 (-10.81)

60 days	0.41±0.01 (-9.89)	0.39±0.02 (-10.95)	7.82±0.34 (-7.69)	6.54±0.30 (-8.91)

90 days	0.41±0.02 (-9.70)	0.42±0.02 (-5.41)	7.86±0.15 (-7.22)	6.72±0.18 (-6.34)

180 days	0.42±0.02 (-7.76)	0.42±0.02 (-5.75)	7.91±0.14 (-6.61)	6.63±0.06 (-7.50)

360 days	0.42±0.00 (-6.96)	0.42±0.00 (-4.05)	7.90±0.12 (-6.77)	6.5±0.11 (-8.43)

p value	<0.01	0.08	0.02	<0.05

^1^Values expressed as mean (standard deviation); ^2^statistical treatment performed using repeated measures ANOVA (p value less than 0.05 was considered significant); ^3^statistical treatment performed using paired t-test and a p value less than 0.05 which was considered significant. AASA: alpha-amino adipic semialdehyde; P6C: piperidine-6-carboxylic acid.

## Data Availability

Readers can access the data underlying the findings upon request to the corresponding author.
